# Sclerosing rhabdomyosarcoma presenting in the masseter muscle: a case report

**DOI:** 10.1186/1746-1596-8-18

**Published:** 2013-02-04

**Authors:** Xu-Yong Lin, Yan Wang, Juan-Han Yu, Yang Liu, Liang Wang, Qing-Chang Li, En-Hua Wang

**Affiliations:** 1 Department of Pathology, the First Affiliated Hospital and College of Basic Medical Sciences, China Medical University, Shenyang 110001, China; 2 Institute of pathology and pathophysiology, China Medical University, Shenyang 110001, China

**Keywords:** Sclerosing rhabdomyosarcoma, Rhabdomyosarcoma, Sarcoma

## Abstract

**Abstract:**

Sclerosing rhabdomyosarcoma (SRMS) is exceedingly rare, and may cause a great diagnostic confusion. Histologically, it is characterized by abundant extracellular hyalinized matrix mimicking primitive chondroid or osteoid tissue. So, it may be easily misdiagnosed as chondrosarcoma, osteosarcoma, angiosarcoma and so on. Herein, we report a case of SRMS occurring in the masseter muscle in a 40-year-old male. The tumor showed a diverse histological pattern. The tumor cells were arranged into nests, cords, pseudovascular, adenoid, microalveoli and even single-file arrays. Immunostaining showed that the tumor was positive for the Vimentin, Desmin and MyoD1, and was negative for CK, P63, NSE, CD45, CD30, S-100, CD99, Myoglobin, CD68, CD34, CD31, and α–SMA. Based on the morphological finding and immunostaining, it was diagnosed as a SRMS. In addition, focally, our case also displayed a cribriform pattern resembling adenoid cystic carcinoma. This may represent a new histological feature which can broaden the histological spectrum of this tumor and also may lead to diagnostic confusion.

**Virtual slides:**

The virtual slide(s) for this article can be found here: http://www.diagnosticpathology.diagnomx.eu/vs/1615846455818924

## Background

Rhabdomyosarcoma (RMS) is relatively uncommon in all soft tissue tumors, and also rare in adults older than 45 years [1]. However, it is the most common soft tissue sarcoma in children. In the current WHO soft tissue tumor classification, RMS is divided into three groups: embryonal (ERMS), alveolar (ARMS) and pleomorphic (PRMS) [1]. The sclerosing rhabdomyosarcoma (SRMS) is an exceedingly rare variant first described by Mentzel and Katenkamp [2], which is still controversial. So far, there are less than 40 reported cases (the present case is not included) in English literatures [3-17]. Because of its rarity, it is still unclear whether SRMS belongs to an unusual subtype of ARMS or ERMS, or even a new variant of RMS [6,8,13,17].

Microscopically, SRMS has a characteristic constellation of features. The neoplastic cells can be arranged into lobules, small nests, microalveoli and even single-file arrays in an abundant hyalinized, eosinophilic to basophilic matrix that closely resembles primitive osteoid or chondroid material [18]. So, it is easily misdiagnosed, if one unfamiliar with the histological spectrum of this entity. Herein, we report a case of sclerosing rhabdomyosarcoma arising in masseter muscle in a 40-year-old male. In addition to the above histological features, the cribriform pattern resembling adenoid cystic carcinoma could also be seen in focal areas. This may represent a new histological feature, which can cause a diagnostic confusion.

## Case presentation

**Figure 1:**
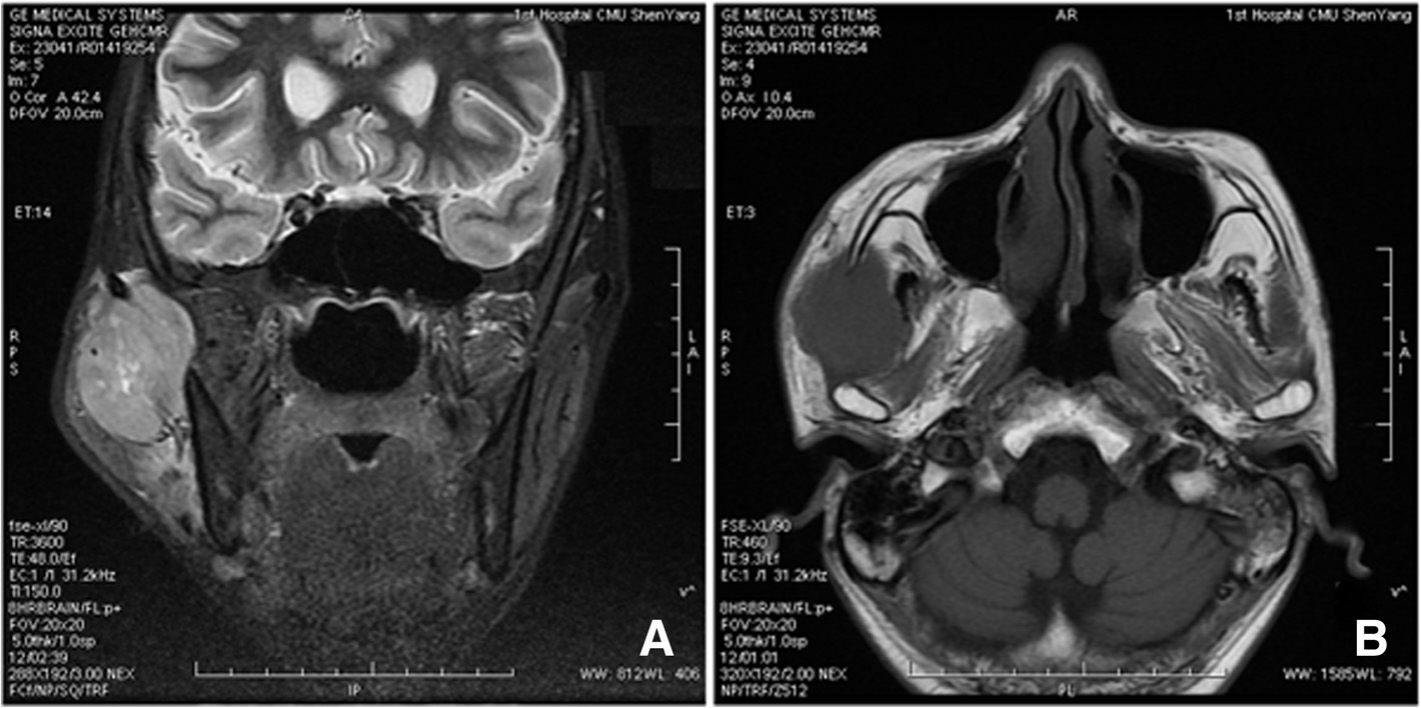
**Magnetic resonance imaging revealing the tumor. A **and **B**, Magnetic resonance imaging revealed an oval mass about 5.2× 3.1 cm in the right lower masseter. The tumor was relatively well circumscribed.

A 40-year-old male presented with a painless swelling in the right parotid gland region. The patient reported the swelling was approximately 1 cm in size 3 months ago, and increased rapidly in size recently. Physical examination revealed a 5-cm subcutaneous mass which felt firm and adhered to the adjacent tissue. Ultrasonography revealed a low echo mass measuring 57.1×26.3 mm in the subcutaneous tissue of the right parotid gland area, the mass was relatively well circumscribed. Magnetic resonance imaging revealed an oval mass about 5.2× 3.1 cm with long T1 and long T2 signal in the right lower masseter (Figure 1). The display of masseter muscle was not clear. The parotid gland and the mandible were compressed, but were not infiltrated by the tumor. Then the tumor was excised and was sent for pathological examination.

## Methods

The submitted specimens were fixed with 10% neutral-buffered formalin and embedded in paraffin blocks. Tissue blocks were cut into 4-μm slides, deparaffinized in xylene, rehydrated with graded alcohols, and immunostained with the following antibodies: Vimentin, Desmin, MyoD1, CK, P63, NSE, CD45, CD30, S-100, CD99, Myoglobin, CD68, CD34, CD31, α–SMA and Ki-67. Sections were stained with a streptavidin-peroxidase system (KIT-9720, Ultrasensitive TM S-P, MaiXin, China). The chromogen used was diaminobenzidine tetrahydrochloride substrate (DAB kit, MaiXin, China), and sections were slightly counterstained with hematoxylin, dehydrated and mounted.

This study was prospectively performed and approved by the institutional Ethics Committees of China Medical University and conducted in accordance with the ethical guidelines of the Declaration of Helsinki.

## Results

**Figure 2:**
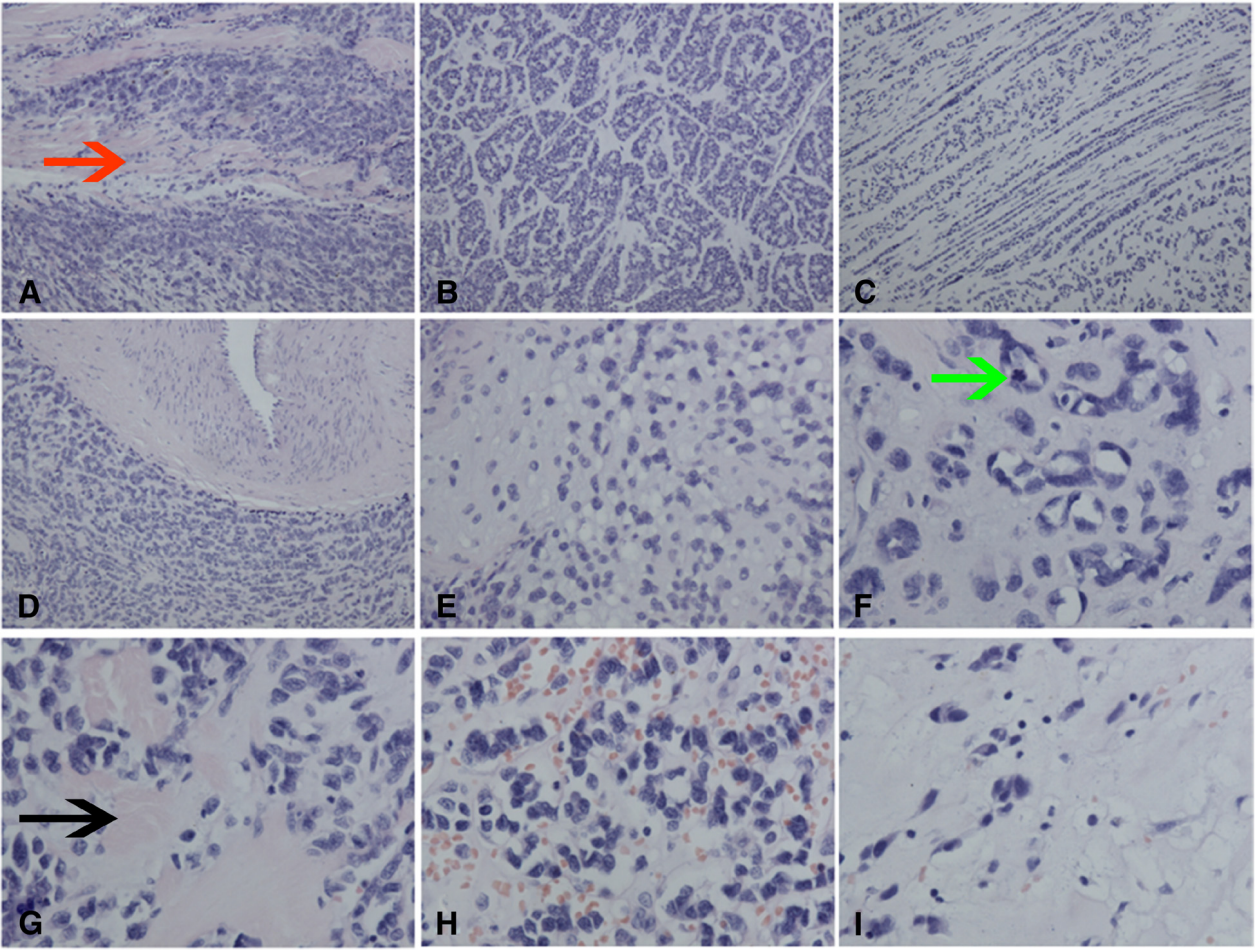
**Morphological changes of the tumor. A**, The tumor infiltrated the normal skeletal muscle tissues (red arrow). **B**, Focally, the tumor cells showed a pattern closely resembling adenoid cystic carcinoma. **C**, The cells were arranged into cords or single-file arrays paralleled with each other. **D**, The cells surrounded the thick-walled blood vessel, and showed a characteristic constellation of features. **E**, Primitive chondroid matrix and cells with intracytoplasmic vacuole could be seen in focal areas. **F**, The tumor cells showed adenoid or microalveoli pattern in abundant basophilic matrix, and an atypical mitosis could be seen (green arrow). **G**, Occasionally, hyalinized, eosinophilic keloid-like collagen (black arrow) could be seen. **H**, Pseudovascular lumens were filled with numerous red cells. **I**, In extensive myxoid stroma, scarcely scattered cells could be seen.

Grossly, the resected tumor measured 5.1×3.0×2.8 cm, and was relatively well circumscribed, the cut surface showed consistently firm and grey-white in colour. Histologically, the tumor displayed a characteristic constellation of features with moderate cellularity, and was predominantly made up of small round and polygonal cells with a small amount of plasma, coarse nuclear chromatin and inconspicuous nucleoli. Focally, the tumor cells showed intracytoplasmic vacuoles resembling chondrocytes. The tumor cells were arranged in a diverse pattern, including nests, cords, pseudovascular, adenoid, microalveoli and even single-file arrays. Moreover, a cribriform pattern resembling adenoid cystic carcinoma could also be seen in focal areas. The mitotic rate of the tumor cells is very high, and atypical mitosis could be seen easily. At the margin, the tumor infiltrated the normal skeletal muscle tissues (Figure 2). In the majority of the whole tumor, the stroma consisted of abundant myxoid or basophilic matrix closely mimicking primitive chondroid materials, and in focal areas, hyalinized eosinophilic keloid-like collagen matrix mimicking primitive osteoid tissues could also be seen. Occasionally, scattered cells could be seen in the extensive myxoid stroma.

**Figure 3:**
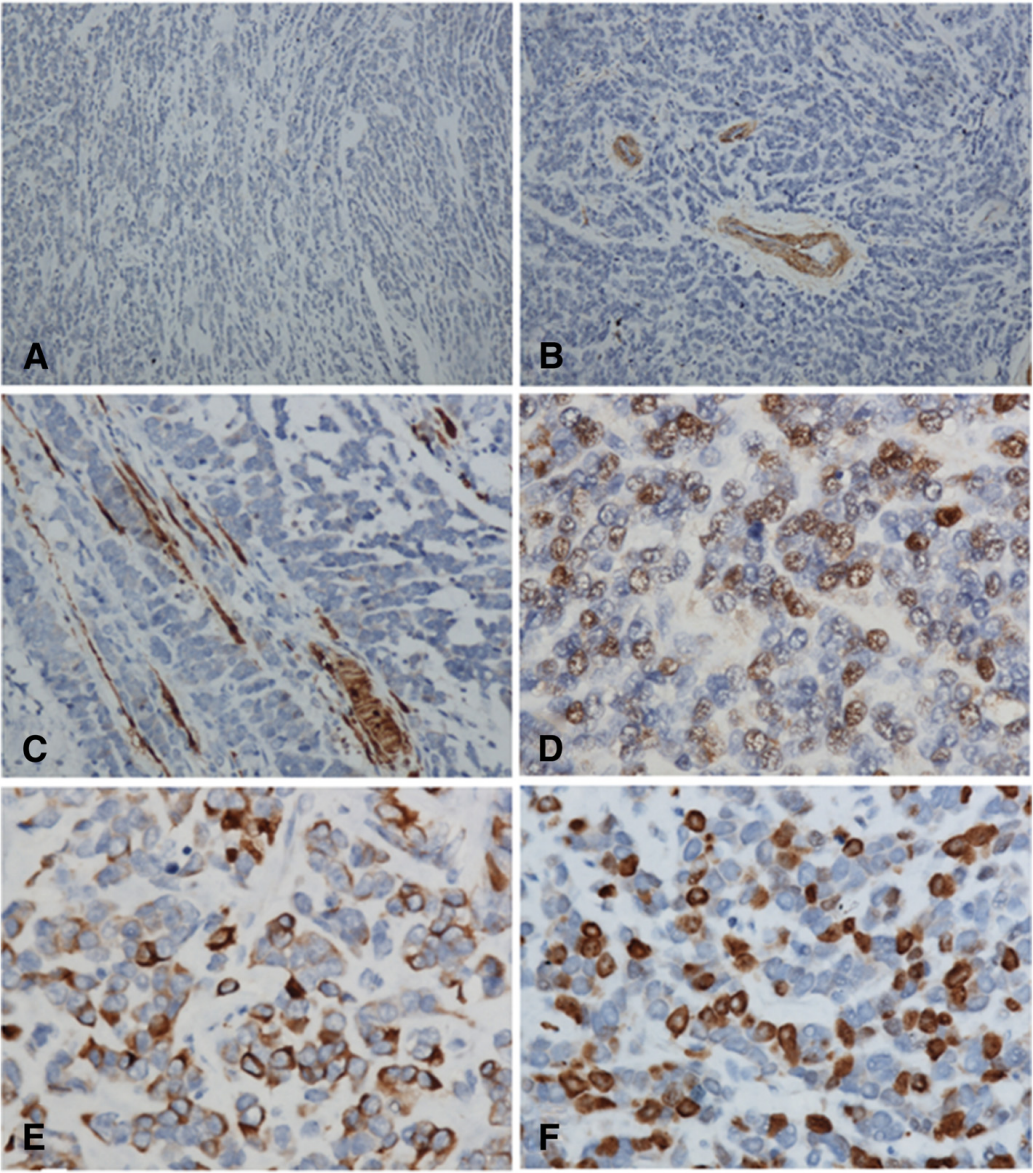
**Immunohistochemical staining for CK, α–SMA, Myoglobin, MyoD1, Desmin and Ki-67 in the tumor. A**, The tumor cells were negative for CK. **B**, α–SMA was expressed in the wall of blood vessel, but not expressed in tumor cells. **C**, Myoglobin immunostaining highlighted the entrapped striated muscle cells, but was not positive in tumor cells. **D**, The tumor cells showed a diffuse positive immunostaining for MyoD1. **E**, The majority of the cells were positive for Desmin. **F**, Ki-67 index was approximate 60%.

Immunostaining showed that the tumor was strongly positive for Vimentin, Desmin and MyoD1, and was negative for CK, P63, NSE, CD45, CD30, S-100, CD99, Myoglobin, CD68, CD34, CD31, α–SMA. Ki67 was expressed in more than 60% of all tumor cells (Figure 3). According to the morphological and immunohistochemical findings, the tumor was diagnosed as a SRMS.

## Discussion

SRMS is an unusual variant of RMS that was first described in 2000 by Mentzel and Katenkamp [2]. They described three cases of RMS in adult patients, characterized by prominent hyaline sclerosis and a pseudovascular growth pattern, and termed sclerosing, pseudovascular rhabdomyosarcoma. In 2002, Folpe *et al.* also described four cases of an unusual hyalinizing, matrix-rich variant of RMS. They named it as sclerosing rhabdomyosarcoma [3]. Subsequently, there have been several additional reports of SRMS. However, so far, there was still less than 40 reported cases (the present case is not included) in English literatures [4-17]. Because of its scarcity, it is still debated whether SRMS is a new variant of RMS or the subtype of ERMS or ARMS. Although SRMS shares some overlapping features with both ERMS and ARMS, it lacks 11p15.5 anomaly frequently observed in ERMS [13,16], and also lacks FOXO1-PAX3 or -PAX7 fusion transcripts associated with ARMS [8]. According to Julie *et al.*, among the 39 reported cases, SRMS can arise in a broad age ranged from 0.3 to 79 years with an average age at 27. The most commonly involved sites (including the present case) are the extremities (19/40) and head and neck (16/40) [17].

Histologically, SRMS has a characteristic constellation of features and is characterized by hyalinized, eosinophilic to basophilic matrix [18]. The tumor usually consisted of small round and polygonal cells with a small amount of plasma, coarse nuclear chromatin and inconspicuous nucleoli. The mitotic rate is very high. The tumor cells were arranged in a diverse pattern, including nests, cords, pseudovascular, adenoid, microalveoli and even single-file arrays. In our case, in a few foci, the tumor cells also displayed a cribiform pattern, which might lead to a diagnostic confusion with adenoid cystic carcinoma. To our knowledge, this is the first reported case which may display the feature resembling adenoid cystic carcinoma.

Immunohistochemically, SRMS is usually strongly positive for Vimentin, Desmin and MyoD1, and weakly, focally positive for Moygenin suggesting its skeletal muscle differentiation, but negative for CK, S-100, CD34, and CD31 [2,3,8,11]. Some cases can also show positive expression of CD99, SMA and CD56 [3,12,17]. In contrast, Myoglobin, a differentiated striated muscle marker was usually not expressed in SRMS, indicating the primitive status of the tumor cell [12,19]. Our immunohistochemical results are generally similar to those reported previously. The tumor cells were strongly positive for Vimentin, Desmin and MyoD1.

The differential diagnosis of SRMS includes osteosarcoma, extraskeletal myxoid chondrosarcoma, mesenchymal chondrosarcoma, sclerosing epithelioid fibrosarcoma, angiosarcoma, parachordoma and even metastatic carcinoma. The typical osteosarcoma is characterized by the presence of matrix calcification, osteoclasts. Extraskeletal myxoid chondrosarcoma typically forms a well circumscribed, multilobulated architecture separated by incomplete fibrous septa. It is composed of round or slightly elongated cells of uniform shape and size usually arranged in short anastomosing strands or cords in myxoid matrix [20]. Mesenchymal chondrosarcoma is characterized by distinct undifferentiated tumor cells admixed with well differentiated cartilagenous components [21]. Sclerosing epithelioid fibrosarcoma is composed of epitheloid cells arranged in nests and cords and deposited in a densely hyalinized collagenous matrix. However, in almost all cases the tumor also shows foci of spindle-shaped sarcoma similar to conventional fibrosarcoma. SRMS focally may also form anastomosing vascular and gland-like spaces mimicing angiosarcoma, but angiosarcoma lacks characteristic hyalinizing matrix of SRMS [22-25]. Parachordoma is typically lobulated and contains nests of vacuolated cells deposited in a myxoid matrix, resembling the physaliphorous cells of chordoma. It usually expresses S-100 protein and CK simultaneously [20]. Moreover, the positive expression of Desmin and MyoD1, negative expression of CK can also rule out the possibility of metastatic carcinoma.

In addition, in our case, the tumor cells focally displayed the cribriform pattern closely mimicking adenoid cystic carcinoma. So, the differential diagnosis may also include the latter. Adenoid cystic carcinoma consists of basaloid cells with round to oval or angulated hyperchromatic nuclei in eosinophilic, hyalinized, or collagenous stroma. Immunohistochemically, adenoid cystic carcinoma can express epithelial cell marker CK, EMA and myoepithelial cell marker P63, S-100 or SMA, by which it can be differentiated from SRMS.

## Conclusion

Because of the rarity, SRMS is misdiagnosed easily, especially if one unfamiliar with this entity. It shows a variable histological pattern. The tumor cells can be arranged into nests, cords, pseudovascular, adenoid, microalveoli and even single-file arrays. In addition, our case also displayed a cribriform pattern resembling adenoid cystic carcinoma in focal areas. To avoid the misdiagnosis, careful attention must be paid to its special histological features.

## Consent

Written informed consent was obtained from the patient for publication of this case report and accompanying images. A copy of the written consent is available for review by the Editor-in Chief of this Journal.

## Competing interests

The authors declare that they have no competing interests.

## Authors’ contributions

**L**XY and WY participated in the histopathological evaluation, performed the literature review, acquired photomicrographs and drafted the manuscript. YJH and LY carried out the immunohistochemical stains evaluation. LQC conceived and designed the study. WL edited the manuscript. WEH gave the final histopathological diagnosis and revised the manuscript. All the authors read and approved the final manuscript.
